# Artificial Intelligence (AI) and Emergency Medicine: Balancing Opportunities and Challenges

**DOI:** 10.2196/70903

**Published:** 2025-08-13

**Authors:** Félix Amiot, Benoit Potier

**Affiliations:** 1 Emergency Department, Service d'Aide Médicale Urgente (SAMU50), Service Mobile d'Urgence et de Réanimation (SMUR), Saint-Lô Memorial Hospital, 715 rue Henri Dunant, Saint-Lô, 50000, France, 33 682640063

**Keywords:** artificial intelligence, artificial general intelligence, large language model, emergency medicine, explainable artificial intelligence, bias, liability, regulation, AI, AGI, LLM, XAI, health data

## Abstract

Artificial intelligence (AI), particularly large language models (LLMs) such as ChatGPT, has rapidly evolved and is reshaping various fields, including clinical medicine. Emergency medicine stands to benefit from AI’s capacity for high-volume data processing, workflow optimization, and clinical decision support. However, important challenges exist, ranging from model “hallucinations” and data bias to questions of interpretability, liability, and ethical use in high-stake environments. This updated viewpoint provides a structured overview of AI’s current capabilities in emergency medicine, highlights real-world applications, and explores concerns regarding regulatory requirements, safety standards, and transparency (explainable AI). We discuss the potential risks and limitations of LLMs, including their performance in rare or atypical presentations common in the emergency department and potential biases that could disproportionately affect vulnerable populations. We also address the regulatory landscape, particularly the liability for AI-driven decisions, and emphasize the need for clear guidelines and human oversight. Ultimately, AI holds enormous promise for improving patient care and resource management in emergency medicine; however, ensuring safety, fairness, and accountability remains vital.

## Introduction

Artificial intelligence (AI), particularly large language models (LLMs) such as OpenAI’s ChatGPT, is experiencing rapid growth in adoption, with a user base of tens of millions on a monthly basis [1]. While the United States and China are making significant advancements in AI technology, Europe is experiencing a lag due to challenges in investment and regulatory frameworks [2,3]. This disparity limits the European public’s comprehension of AI’s potential applications and implications.

In medicine, AI demonstrates exceptional capabilities in processing complex health data [4], advanced medical imaging [5,6], anatomopathology [5], and electrocardiogram interpretation [6]. Furthermore, AI exhibits proficiency in “internship competitions” [7] and has the potential to surpass practitioners in medical diagnoses [8]. AI significantly contributes to medical research by aiding the creation of scientific articles [9] and advancing new therapeutic areas, especially in infectiology [10].

Nevertheless, it is posited that there is an underestimation of the substantial impact of this technology on contemporary and future medicine. Recent advancements in AI, propelled by extensive computational power rather than conceptual innovations, demonstrate that the potential of these systems depends primarily on the accumulation of data, duration of training, and financial resources [11]. This observation suggests that the improvement of AI systems necessitates an increase in high-quality data, extended training periods, and financial investment, rather than relying solely on the ingenuity of researchers.

Emergency medicine is a high-stakes field in which rapid and accurate decision-making is crucial. Overcrowded departments, resource constraints, and time pressure create significant challenges that advanced AI can mitigate. However, the complexity and unpredictability of emergency medicine highlight the importance of safety, liability, and ethical considerations. This paper presents the current applications of AI in emergency medicine, emphasizing both real-world implementations and critical challenges, such as hallucination, bias, and interpretability.

## Current AI Capabilities in Emergency Medicine

AI systems, particularly deep learning networks, can be used to identify patterns in large, complex datasets, significantly contributing to medical science by analyzing variables to reliably predict outcomes [12,13]. In emergency departments (EDs), this enables improved triage, patient flow management, and resource allocation in overcrowded settings where resources are limited and time is critical [14-19]. Hospitals are also investigating AI’s potential to optimize resource allocation by forecasting bed availability and scheduling staff [20,21]. In one study, an AI tool analyzing historical admission patterns, vital signs, and laboratory data enhanced ED throughput by identifying high-risk patients for earlier admission [22].

Moreover, AI has demonstrated high accuracy in clinical tasks such as the recognition of acute coronary syndromes, recognition of acute appendicitis, and rapid interpretation of imaging for fractures or head injuries [23-30]. These tasks involve pattern recognition in large data streams—vital signs, laboratory results, or imaging scans—and can reduce diagnostic delays. Some EDs have tested AI models for specific conditions, such as early sepsis detection, by monitoring the vital signs and laboratory results [31]. This approach has shown promise in reducing the time to antibiotic administration, although the results vary across different populations.

Conventional clinical decision support systems (CDSSs) have been widely used in health care settings to assist medical professionals in making informed decisions. These systems typically operate on the basis of predefined rules, scoring systems, or algorithms developed by experts in the field. Although effective in many scenarios, these traditional CDSSs have limitations in their ability to adapt to new information or complex, multifaceted clinical situations [32]. In contrast, AI models, particularly LLMs, represent a paradigm shift in clinical decision support. These models leverage vast amounts of data to learn patterns and associations without the need for explicit rule-coding [33,34]. This data-driven approach allows AI models to potentially identify subtle or complex relationships within medical data that may be overlooked by traditional CDSSs or even by human experts [35]. However, it introduces challenges in interpretability as the “reasoning” behind AI outputs may be opaque.

Although these early successes are encouraging, robust prospective trials remain limited. Further evidence from large-scale, peer-reviewed studies is necessary to confirm whether AI-driven solutions consistently improve patient outcomes in emergency care settings.

## Challenges and Limitations

A widely documented phenomenon in LLMs is “hallucination,” where the model confidently generates inaccurate or fabricated information [36-38]. While all diagnostic tools are susceptible to errors, AI hallucinations can be especially problematic because they are delivered convincingly, making them difficult to spot. In emergency settings, where time constraints are acute, misleading AI outputs can significantly affect patient care. The current research highlights the need for rigorous validation of LLM outputs, particularly when used for direct patient management. Because AI models are trained on large datasets, their performance is strongest for common presentations. Infrequent conditions such as rare genetic disorders or atypical manifestations of common pathologies are prone to misclassification. Moreover, LLMs struggle with rare and atypical cases that are common in emergency medicine, making them less effective in unconventional clinical situations. This limitation arises because LLMs rely on statistical correlations, favoring common cases over unique ones [39]. Additionally, LLMs are not programmed to indicate uncertainty, which increases the risk of misleading information in critical situations [40,41].

Data-driven models of emergency care can significantly improve patient outcomes and operational efficiency. However, their reliance on historical data can perpetuate the existing biases, leading to unequal treatment across diverse patient populations. This is concerning in emergency settings, where rapid decision-making is crucial and biased algorithms can be life-threatening. An AI system trained predominantly on data from one ethnic group might misinterpret the symptoms presented by patients from other backgrounds, resulting in delayed or inappropriate care. A study using an AI-powered dermatological algorithm clearly demonstrated this problem. When applied to Fitzpatrick skin type 6 (dark skin) dermatological conditions, the AI system achieved only 17% diagnostic accuracy compared to 69.9% for Caucasian skin types [42]. Interestingly, AI systems can also be designed to detect limitations when faced with unfamiliar data. Conformal prediction techniques have been shown to flag unreliable predictions when an AI system encounters new data from different laboratories, scanners, or pathology assessments [43]. This approach could help mitigate risks associated with AI systems, such as incorrect diagnoses for patients from underrepresented groups. Diversifying training datasets is critical to ensure that AI models are exposed to a wide range of patient demographics and clinical presentations. Implementing fairness-aware algorithms that consider and adjust for potential biases during decision-making is equally important. Continuous monitoring and auditing of AI system performance across demographic subgroups is essential for identifying and rectifying emerging disparities. This vigilance, combined with regular updates to models and training data, can help ensure that AI-driven emergency care systems serve all patients equally, regardless of their background or socioeconomic status.

## Model Interpretability and Explainable AI (XAI)

AI-based triage systems in emergency medicine are powerful and complex tools for optimizing patient care. The “black-box” nature of these algorithms raises ethical concerns in high-stakes emergency settings, where rapid and accurate decision-making is crucial [44]. When an opaque AI system designates a patient as “low priority” without clear justification, it may lead to prolonged wait times or reduced attention, potentially compromising patient outcomes.

Explainable artificial intelligence (XAI) is important in emergency medicine. Transparent and interpretable AI models are essential to maintain trust, accountability, and safety in emergency care [45]. XAI techniques such as Shapley Additive Explanations or Local Interpretable Model-Agnostic Explanations can provide insights into which variables most influence a model’s output, making AI-based recommendations more transparent to health care professionals [46]. This interpretability is crucial in emergency settings, where clinicians must quickly understand and validate AI recommendations.

In the fast-paced environment of emergency medicine, the balance between model performance and explainability is critical. Although complex models may offer superior predictive power, their opacity can hinder clinical trust and integration [47]. Developing AI systems that maintain high accuracy while providing clear explanations for their decisions is an active research area, particularly relevant to emergency care, where trust and clarity are paramount [48].

To address these challenges, transparent and XAI triage mechanisms tailored to emergency medicine are crucial. These systems should provide immediate insights into the decision-making process, allowing health care professionals to quickly understand and validate AI recommendations in time-sensitive situations. Structured override processes must be established to enable rapid human intervention, when necessary, ensuring that algorithmic decisions can be reviewed and adjusted based on clinical judgment and contextual factors.

By prioritizing transparency, explainability, and human oversight in AI-based triage systems for emergency medicine, health care institutions can harness AI benefits, while maintaining fairness, accountability, and public trust. This approach enhances patient care quality, supports clinician confidence, facilitates training, and helps to identify potential failure modes in critical care situations.

## The Evolving Regulatory Landscape

Comparative analyses of the European Union, United States, and international regulatory frameworks reveal distinct policy priorities. The European Union’s Artificial Intelligence Act and General Data Protection Regulation established a structured, risk-based approach for health care AI, mandating transparency, accountability, and bias prevention [2,49]. The United States relies on sector-specific regulations, notably the Food and Drug Administration’s Total Product Life Cycle framework for Software as a Medical Device, prioritizing flexible risk stratification to accommodate continuous AI model updates with weaker postmarket monitoring [50,51]. International bodies, including the United Nations Educational, Scientific and Cultural Organization, the World Health Organization, and the Organisation for Economic Co-operation and Development, have advanced frameworks for ethical AI governance—centering on fairness, transparency, and human oversight—but lack enforceable mechanisms, limiting regulatory convergence across regions [52].

Health care providers face liability concerns when AI-driven recommendations lead to misdiagnoses or suboptimal treatment. The unpredictable nature of AI systems is complex. As legal frameworks evolve, questions arise about responsibility when errors stem from “black-box” AI. Should providers be held accountable for following AI advice? Can liabilities extend to AI developers or institutions? Jurisdictions worldwide are addressing these issues [46,53]. The European Union’s proposed Artificial Intelligence Act [2] and the US Food and Drug Administration’s guidance on AI in medical devices represent initial steps [50], but specific guidelines for LLM-driven decision support remain limited. The global health care community recognizes the need for rigorous validation and clear risk stratification before integrating AI tools into clinical workflows [54]. Regulatory bodies emphasize robust clinical validation, decision-making traceability, and mechanisms to update AI tools as new data emerge. However, formalized legal precedents for AI liabilities in emergency medicine are still emerging. This lack of established guidelines creates uncertainty for the stakeholders. As AI is integrated into medical practice, stakeholders must collaborate to develop comprehensive frameworks that balance innovation with patient safety and legal protection, addressing transparency, ongoing monitoring, and clear delineation of responsibilities [53,54].

A pressing issue is the global disparity in the adoption of AI regulations. While the European Union has implemented binding legal frameworks and the United States is adapting existing structures to AI technologies, a significant regulatory gap persists in many regions; only 15.2% of countries have enacted AI-specific health care regulations, amplifying concerns over global inequities in AI development and patient safety [55]. Scholars have increasingly advocated hybrid approaches that integrate risk-based innovation enablement with enforceable ethical guardrails to bridge the regulatory divide between EU prescriptive models, US flexible guidelines, and international voluntary standards.

## Toward Responsible and Fair AI Implementation

EDs serve diverse patient populations, necessitating a focus on fairness. Institutions employ several strategies to mitigate bias.

First, diversifying the training data ensures representation across demographics, locations, and disease phenotypes. Second, the implementation of fairness-aware algorithms adjusts model parameters to reduce performance disparities. Third, conducting regular audits helps to monitor AI outputs for patterns of discrimination.

While AI offers powerful capabilities, it should complement, rather than replace, clinical judgment. Humans excel in contextual understanding, ethical reasoning, and handling unique cases, whereas AI excels in recognizing patterns in large datasets. A collaborative approach combining AI recommendations with clinician reviews can help to identify errors or biases. Some institutions have established AI oversight committees to evaluate the performance, manage updates, and review near-miss incidents.

To address the risks of AI hallucinations, institutions should implement structured verification processes for AI-suggested diagnoses or treatments. Encouraging AI models to express confidence levels or flag uncertain outputs through uncertainty quantification is beneficial. Additionally, requiring critical decisions prompted by AI to be documented with explanations can increase trust.

AI-driven triage can expedite care, but it also raises questions about distributive justice and patient autonomy. Transparency in how AI models prioritize patients is crucial, and ED staff should retain the authority to override AI-driven triage decisions when necessary.

## Future Directions and Conclusion

The integration of AI into emergency medicine holds tremendous potential to revolutionize clinical practice, from optimizing patient flow to aiding in complex diagnoses. However, these powerful tools are associated with significant risks and require careful governance. Ensuring model interpretability through XAI is crucial for building trust and validating clinical applications. Unresolved liability issues necessitate collaboration between health care providers, policy makers, and AI developers to clearly define their responsibilities.

Moving forward, researchers and ED administrators should prioritize several key areas:

1. Comprehensive, multisite validation: large-scale prospective studies should be conducted to assess the real-world impact of AI on patient outcomes and safety.
2. Robust regulatory frameworks: clear guidelines addressing liability, performance monitoring, and transparency requirements should be developed.
3. Bias mitigation: fairness-aware techniques and regular audits should be implemented to ensure equitable treatment across diverse patient populations.
4. Educational initiatives: clinicians, residents, and administrators should be trained to use AI responsibly, critically interpret outputs, and maintain essential diagnostic skills.

The rapid evolution of AI underscores its transformative potential and the need for prudent caution. By implementing appropriate safeguards across the technical, legal, ethical, and educational domains, AI can become a powerful ally in emergency medicine, ultimately enhancing patient care and outcomes.

## Abbreviations

AI: artificial intelligence
CDSS: clinical decision support system
ED: emergency department
LLM: large language model
XAI: explainable artificial intelligence
